# Young Individuals Are More Stable and Stand More Upright When Using Rollator Assistance During Standing up and Sitting Down

**DOI:** 10.3389/fbioe.2022.876349

**Published:** 2022-07-11

**Authors:** F. C. Krafft, M. Herzog, T. Stein, L. H. Sloot

**Affiliations:** ^1^ Optimization, Robotics, and Biomechanics (ORB), Institute of Technical Engineering (ZITI), Heidelberg University, Heidelberg, Germany; ^2^ HEiKA—Heidelberg Karlsruhe Strategic Partnership, Karlsruhe Institute of Technology (KIT), Heidelberg University, Heidelberg, Germany; ^3^ BioMotion Center, Institute of Sports and Sports Science (IFSS), Karlsruhe Institute of Technology (KIT), Karlsruhe, Germany

**Keywords:** assisted movement, center of pressure, kinematics, rollator, sit-to-stand, stand-to-sit

## Abstract

Four-wheeled walkers or rollators are often used to assist older individuals in maintaining an independent life by compensating for muscle weakness and reduced movement stability. However, limited biomechanical studies have been performed to understand how rollator support affects posture and stability, especially when standing up and sitting down. Therefore, this study examined how stability and posture change with varying levels of rollator support and on an unstable floor. The aim was to collect comprehensive baseline data during standing up and sitting down in young participants. In this study, 20 able-bodied, young participants stood up and sat down both 1) unassisted and assisted using a custom-made robot rollator simulator under 2) full support and 3) touch support. Unassisted and assisted performances were analyzed on normal and unstable floors using balance pads with a compliant surface under each foot. Using 3D motion capturing and two ground-embedded force plates, we compared assistive support and floor conditions for movement duration, the relative timing of seat-off, movement stability (center of pressure (COP) path length and sway area), and posture after standing up (lower body sagittal joint angles) using ANOVA analysis. The relative event of seat-off was earliest under full support compared to touch and unassisted conditions under normal but not under unstable floor conditions. The duration of standing up and sitting down did not differ between support conditions on normal or unstable floors. COP path length and sway area during both standing up and sitting down were lowest under full support regardless of both floor conditions. Hip and knee joints were least flexed under full support, with no differences between touch and unassisted in both floor conditions. Hence, full rollator support led to increased movement stability, while not slowing down the movement, during both standing up and sitting down. During standing up, the full support led to an earlier seat-off and a more upright standing posture when reaching a stable stance. These results indicate that rollator support when handles are correctly aligned does not lead to the detrimental movement alterations of increased forward-leaning. Future research aims to verify these findings in older persons with stability and muscle weakness deficiencies.

## Introduction

Movement instabilities that lead to falls are a major problem for older individuals during activities of daily living (Rapp et al., 2012; Center for Disease Control and Prevention 2020). The traumatic incident and the associated injuries limit their ability to live independently, increasing the risk of disability, institutionalization, and mortality, therby affecting the overall quality of life (Guralnik et al., 1994; Lindemann et al., 2007; Ambrose et al., 2013; Geravand et al., 2017). To overcome instabilities and muscular weakness, four-wheeled walkers, also known as rollators, are often prescribed to maintain daily life mobility (Löfqvist et al., 2007; Geravand et al., 2017; Costamagna et al., 2019). However, recent findings also assume that rollators do not generally prevent falling and can even contribute to an increased fall risk (Hefflin et al., 2004; Deandrea et al., 2010).

So far, not much is known about the effect of rollator assistance on balance and posture during gait. Studies that examined the use of assistive devices during walking indicate that assisted walking leads to a more bent body position, with a greater forward tilt of the trunk (Carey and Crompton 2005; Drzał-Grabiec et al., 2013) and increased hip and knee flexion (Boyer et al., 2017) compared to unassisted walking. This forward-leaning strategy enables load transfer of the trunk *via* the arms to the assistive device and has been suggested to increase stability by creating a larger base of support (Bateni and Maki 2005; Costamagna et al., 2019). Furthermore, increased muscular co-contraction and co-activation lead to increased flexion in the legs’ joints and thus enhanced joint stiffness and movement stability (Bouchouras et al., 2015). However, sagittal plane stiffening might not translate to sufficient stability in situations of medio-lateral perturbations and instabilities. Therefore, such alterations of posture might well reduce the body’s capacity to compensate for situations that lead to falls, with a sub-optimal positioning of the person relative to the rollator, dependency on fatiguing arm muscles, and over time, a potential prolonged lower limb unloading that could deteriorate lower limb weakness (Costamagna et al., 2019). However, individuals who use the assistive device as a balance aid only can have enhanced movement stability compared to individuals who fully lean on the assistive device by providing spatial information to the central nervous system by touching the handles (Jeka et al., 1996; Jeka 1997; Dickstein et al., 2003; Martinelli et al., 2015; Costamagna et al., 2019; Komisar et al., 2019; Oates et al., 2020). Therefore, it remains unclear how to optimally use a rollator for optimal movement stability and loading support.

Furthermore, there is a lack of knowledge of how rollators change movement during different tasks. For instance, how posture is influenced by an assistive device in standing up and sitting down and how changes in posture, in turn, influence movement stability (Mundt et al., 2019).

Standing up and sitting down are two of the most frequently performed movements in daily life. These movements are of high relevance for independent living because they are a major prerequisite for upright posture, which is essential for gait initiation and other activities of daily living (Galli et al., 2008; Lee et al., 2015; Bobbert et al., 2016; Duarte Wisnesky et al., 2020). However, standing up and sitting down are among the most affected activities in older individuals (Sütçü et al., 2019), and because of that, they represent the movements in which fall events most frequently occur in frail, older individuals (Rapp et al., 2012). However, there is still a lack of knowledge about the reasons that lead to falls during standing up and especially during sitting down and whether assistive support increases or decreases movement stability.

Successful performance of standing up requires a complex interplay of trunk motion and leg joint coordination to safely transfer the body to a stable upright position and to realize safe seating during sitting down. To realize seat lift-off, body weight has to be shifted anteriorly over the feet by forward trunk flexion, followed by a momentum transfer to move the body in a vertical direction. The vertical lift is dominated by simultaneous hip, knee, and ankle extensions and is finalized by a stabilization phase executed and maintained by the legs’ musculature (Schenkman et al., 1990; Millington et al., 1992; Roebroeck et al., 1994; Papa and Cappozzo 2000; Scarborough et al., 2007; Bohannon 2015). However, this complex interplay of trunk motion and leg joint coordination bears the potential of failure in older individuals because of their reduced physical capacities. For instance, adequate range of motions and torque generation in the leg joints are often impaired in older individuals (Rodosky et al., 1989; Bouchouras et al., 2015). This mechanism leads to higher joint stiffness (Solomonow et al., 1987; Baratta et al., 1988) and an altered control of the force transfer from the hip to the knee joint (Roebroeck et al., 1994). Thus, it is not fully investigated how the support of an assistive device provides the best movement stability during assisted standing up and sitting down in the light of these common aging processes, nor in younger participants, which is important for fundamental understanding of provided support on stability.

Therefore, the purpose of this study was to perform a comprehensive analysis of balance and posture during both standing up and sitting down while receiving the support of an assistive device in a group of young, able-bodied participants. For a comprehensive assessment of different effects of support, we analyzed full weight support by leaning on the handles versus the stabilization proprioceptive input obtained via a touch of the handles versus no assistance in terms of movement speed, stability, and posture. To examine the effect of support in more balancing challenging conditions, we also analyzed the standing up and sitting down movements on unstable floor conditions. To provide a broad analysis of the movements, from a spatiotemporal perspective, the duration of the standing up and sitting down movement was analyzed as prolonged standing up and sitting down are associated with fall risk in, e.g., clinical five times chair rise test (Guralnik et al., 1994; Cheng et al., 2014). Furthermore, as the preparation process before lifting the buttocks of the chair is highly important for the stability of the subsequent standing up movement (Goulart and Valls-Solé 1999), we analyzed the relative seat-off timing to quantify if the variations in support led to changes in the preparation time. Because the center of pressure (COP) reflects the neuromuscular response to control the center of mass (COM) within the base of support, it has an important role in terms of maintaining stability (Sloot et al., 2020; Richmond et al., 2021). Therefore, we analyzed throughout the standing up and sitting down the total 2D horizontal COP (COP_feet_) path length of the feet (Pinsault and Vuillerme 2009; Ringhof and Stein 2018) and the sway area (Quijoux et al., 2021). Furthermore, as rollators can lead to a hunched body position that is described as detrimental for overloading the upper body and building a rather unstable body position (Carey and Crompton 2005; Drzał-Grabiec et al., 2013; Boyer et al., 2017), we analyzed the body posture at the end of the standing up movement to detect if the variations in support also lead to the posture variations that are described in walking. The end posture position is assumed to reflect the starting position when initiating gait after standing up.

We hypothesized that 1) standing up and sitting down durations are reduced and seat-off preparation time is reduced during assistive support conditions compared to the unassisted performance; 2) both support conditions lead to improved stability under normal and unstable floor conditions; and 3) hip, knee, and ankle joints are more flexed at a stable stance under full support compared to light touch and unassisted support conditions because of leaning.

## Methods

### Participants

In this study, 20 able-bodied individuals [10 women (23.9 ± 3.4 years) and 10 men (27.9 ± 5.8 years)] voluntarily participated in the study. The study design was approved by the Ethics Committee of the Medical Department of Heidelberg University (S-105/2021). All participants gave their written informed consent before study participation.

### Experimental Setup and Protocol

**FIGURE 1:**
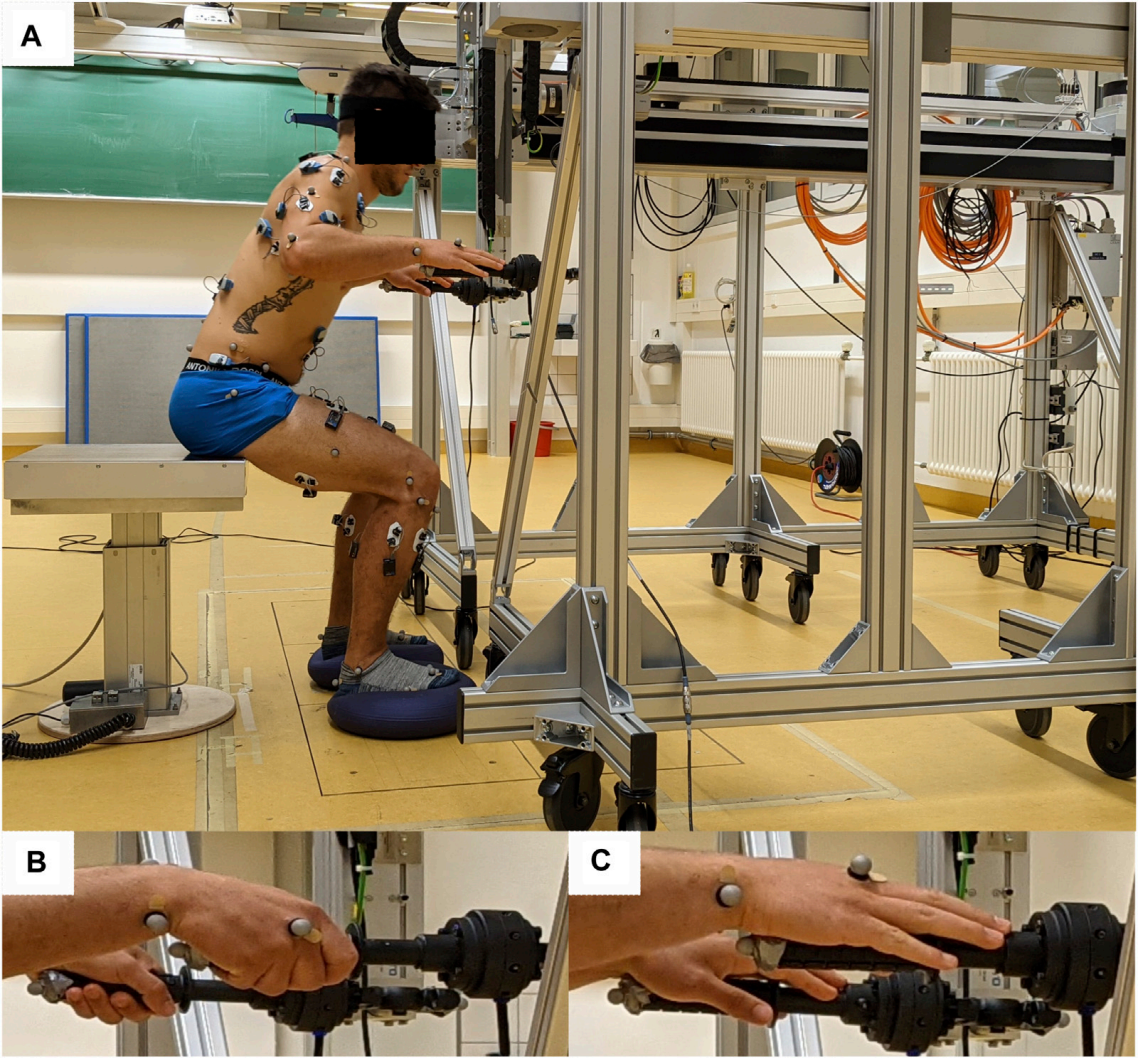
Experimental setup. **(A)** Male participant standing up with the custom-made robot rollator simulator. A balance-pad was used under each foot to induce unstable floor conditions during standing up and sitting down. Full-body passive markers for motion tracking and EMG electrodes (data not included in this article) were placed on the body. **(B)** Hand position during full support condition. **(C)** Hand position under touch condition.

The participants stood up and sat down at their preferred speed with the instruction: “stand up, stand still, sit down.” Three different support conditions were tested using a custom-made robot rollator simulator with instrumented, height-adjustable handles (Figure 1A). The support conditions included the following: 1) unassisted; 2) full support, wherein the participants were instructed to fully grab the rollator handles with a power grip to receive maximal support and to fully lean onto the rollator handles (Figure 1B); and 3) touch, wherein the hands were placed with a palm grip onto the rollator handles to receive a stabilizing proprioceptive input in terms of a haptic cue (Figure 1C). During full support and touch conditions, the handle height was individually adjusted to the height of the wrist, specifically the distal radius head (i.e., processus styloideus radii), measured when the arms were hanging down in a standing position. At the beginning of the standing up movement, the handles were positioned in a way that the distal ends of the handles were leveled to the toe markers in the anterior–posterior direction. This position allowed the participants to pull themselves up with the support of the handles. At the end of the movement, the handles were held sideways at the body. The unstable floor condition was created by a circular rubber-made balance-pad with a compliant surface (Dynair^®^ Ballkissen^®^, diameter 33 cm, height 8 cm, TOGU GmbH, 83209 Prien-Bachham, Germany) placed underneath each foot (Figure 1A) and compared to the normal floor condition. The order of support and floor conditions were randomized for each participant. The participants performed two familiarization trials for each support and floor condition to ensure safe and valid performance. All participants had to perform three valid trials of standing up and sitting down, with trials repeated when they were not continuous throughout the transition from seating to stable standing and vice versa and had, for instance, any compensatory movement of the arms or the feet (e.g., side-stepping). This resulted in a sum of 18 valid standing up and sitting down trials for each participant. Trials started and ended with the arms hanging on their sides, and no further conditions on arm movement were given for the unassisted condition. Seat height was adjusted to the knee height for each participant.

### Data Acquisition and Post-processing

Force data were collected using two ground-embedded 3D force plates (1,000 Hz; Bertec Corp., Columbus, OH, United States), with each foot standing on a force plate (normal floor condition) or a balance-pad that was placed on each force plate separately (unstable floor condition). Forces applied to the rollator simulator were collected using a 3D force measuring sensor embedded into each handle (100 Hz; Robotiq Inc., Lévis, QC, Canada). In addition, loading of the chair was measured using four force sensors integrated into the seating surface (1,000 Hz; Phidgets Inc., Calgary, AB, Canada). Movement data were simultaneously collected using a passive 3D motion capture system at 150 Hz (10 type 5 + cameras, Qualisys, Gothenburg, Sweden). Spherical reflective motion capture markers (14 mm) were placed on anatomical landmarks of each participant according to the IOR full-body marker model (Cappozzo et al., 1995; Leardini et al., 2011), with additional iliac crest and greater trochanter markers to ensure tracking of the pelvis and thighs throughout the whole movement.

After recording, motion capturing data were post-processed (3D reconstructed, labeled, and gap-filled) with Qualisys Tracking Manager (version 2018.1, Qualisys). Thereafter, full-body kinematics, including the COM, were calculated using the standard IOR full-body human model in Visual3D (version 6, C-Motion Inc., Germantown, MD, United States). Data were filtered with a bi-directional 10 Hz low-pass 4^th^ order Butterworth filter.

Data segmentation for the definition of the net standing up and sitting down phases was conducted in Matlab (version R2020a, Natick, MA, United States). To consistently identify the times of movement initiation and ending, we used a clustering approach to consider movement variability while not needing artificial thresholds (Sloot et al., 2020). We used the k-means++ algorithm to cluster both the resultant COM velocity in the anterior–posterior and vertical direction and the COM height into three clusters each. The start of standing up (or end of sitting down) was defined as the end of the period with low COM velocity as well as low COM height, and the end of standing up (or the start of sitting down) was the start of the period of low COM velocity with high COM height clusters. The moments of seat-off and seat-on were derived from the unfiltered force data measured by the force sensors in the seat of the instrumented chair. For the four participants who did not have these data, the ground reaction forces measured under the feet, alongside marker and COM data were used to determine these events. After visually checking segmentation, motion and force data were time normalized to 100% standing up and sitting down movement durations.

### Dependent Variables and Statistics

To assess the effect of support and floor condition on movement stability and body posture, the following dependent variables were calculated: 1) movement speed in terms of the total duration of standing up and sitting down and the relative event of seat-off or seat-on; 2) stability in terms of the total 2D horizontal COP_feet_ path length averaged over both legs (Pinsault and Vuillerme 2009; Ringhof and Stein 2018) and 95% confidence ellipse area (sway area) averaged over both legs from seat-off to a stable stance and vice versa (Quijoux et al., 2021); and 3) posture in terms of sagittal plane angles at a quiet stance in the hip, knee, and ankle joint averaged over both legs. Descriptive data are presented as means and standard deviations for the dependent variables. One-way repeated measures ANOVAs with the factor *support* (full support, touch, and unassisted) were computed for each of both floor conditions (standard and unstable) using SPSS statistics (version 27, IBM, Armonk, NY, United States). Kolmogorov–Smirnov and Mauchly’s tests were used to confirm the normality and sphericity of the data distribution, respectively. If the ANOVA showed an effect of support condition, pairwise *t*-tests for dependent samples were computed as post hoc tests between the support conditions with Holm–Bonferroni corrections for multiple comparisons (Holm 1979). The level of significance for all statistical tests was set *a priori* at *p* ≤ 0.05. The effect size Cohen’s d was calculated for the pairwise t-tests for dependent samples. According to Cohen (1992), large effects are indicated by *d* = 0.8, medium effects by *d* = 0.5, and small effects by *d* = 0.2.

## Results

### Influence of Support on Movement Duration and Seat-Off and Seat-On Events

There were no significant differences in the influence of the support conditions on the duration of standing up (*p* = 0.65) and sitting down (*p* = 0.86) on the normal or unstable floor (standing up: *p* = 0.25; sitting down: *p* = 0.27). The total durations during standing up on the normal floor were the following: unassisted, 1.21 ± 0.17 s; touch, 1.22 ± 0.20 s; and full support, 1.25 ± 0.27 s. During sitting down on the normal floor, the total durations were the following: unassisted, 1.43 ± 0.23 s; touch, 1.41 ± 0.27 s; and full support, 1.45 ± 0.24 s. During standing up on the unstable floor, the total durations were the following: unassisted, 1.36 ± 0.33 s; touch, 1.27 ± 0.20 s; and full support, 1.25 ± 0.27 s. During sitting down on the unstable floor, the total durations were the following: unassisted, 1.44 ± 0.26 s; touch, 1.38 ± 0.23 s; and full support, 1.45 ± 0.24 s.

**FIGURE 2:**
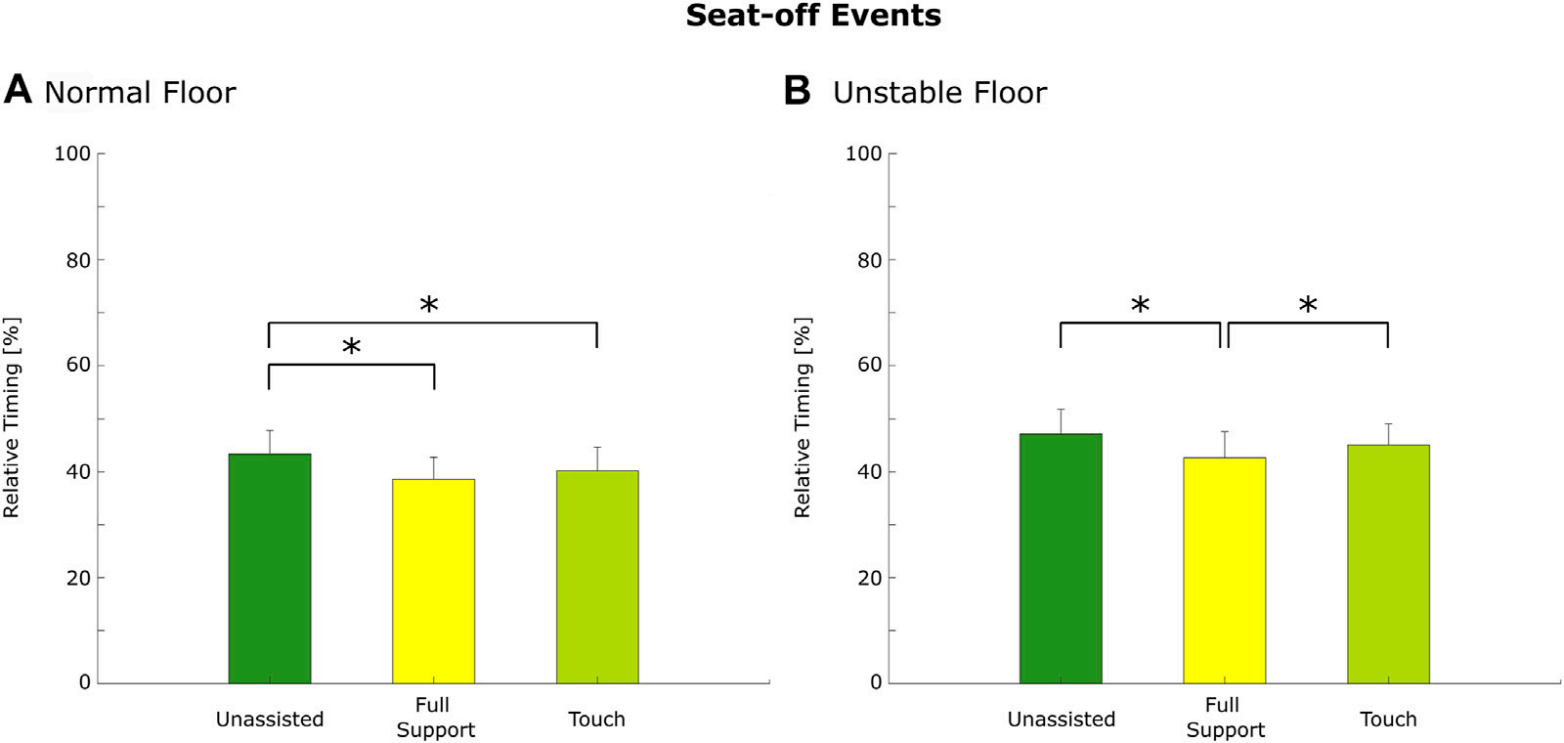
The relative timing of seat-off on normal **(A)** and unstable **(B)** floors. The bar represents the mean value, the error bar represents the standard deviations, and the asterisk depicts post hoc significant differences with *p* ≤ 0.05 (Holm–Bonferroni corrected).

However, the relative timing of seat-off was affected by the support conditions on normal (*p* < 0.01) and unstable (*p* < 0.01) floor conditions, whereas seat-on was not affected by the support conditions on the normal (*p* = 0.70) or unstable (*p* = 0.17) floor. During standing up on the normal floor, seat-off was significantly later in the unassisted condition (43.4 ± 4.4%) compared to full support (38.6 ± 4.2%; *p* < 0.01; *d* = 1.11) and to touch (40.2 ± 4.5%; *p* < 0.01; *d* = 0.72; Figure 2A). During standing up on the unstable floor, seat-off was significantly later under touch (45.0 ± 4.0%; *p* < 0.01; *d* = 0.52) and unassisted (47.1 ± 4.7%; *p* < 0.01; *d* = 0.93) compared to the full support condition (42.6 ± 5.0%; Figure 2B).

### Influence of Support on Movement Stability

**FIGURE 3:**
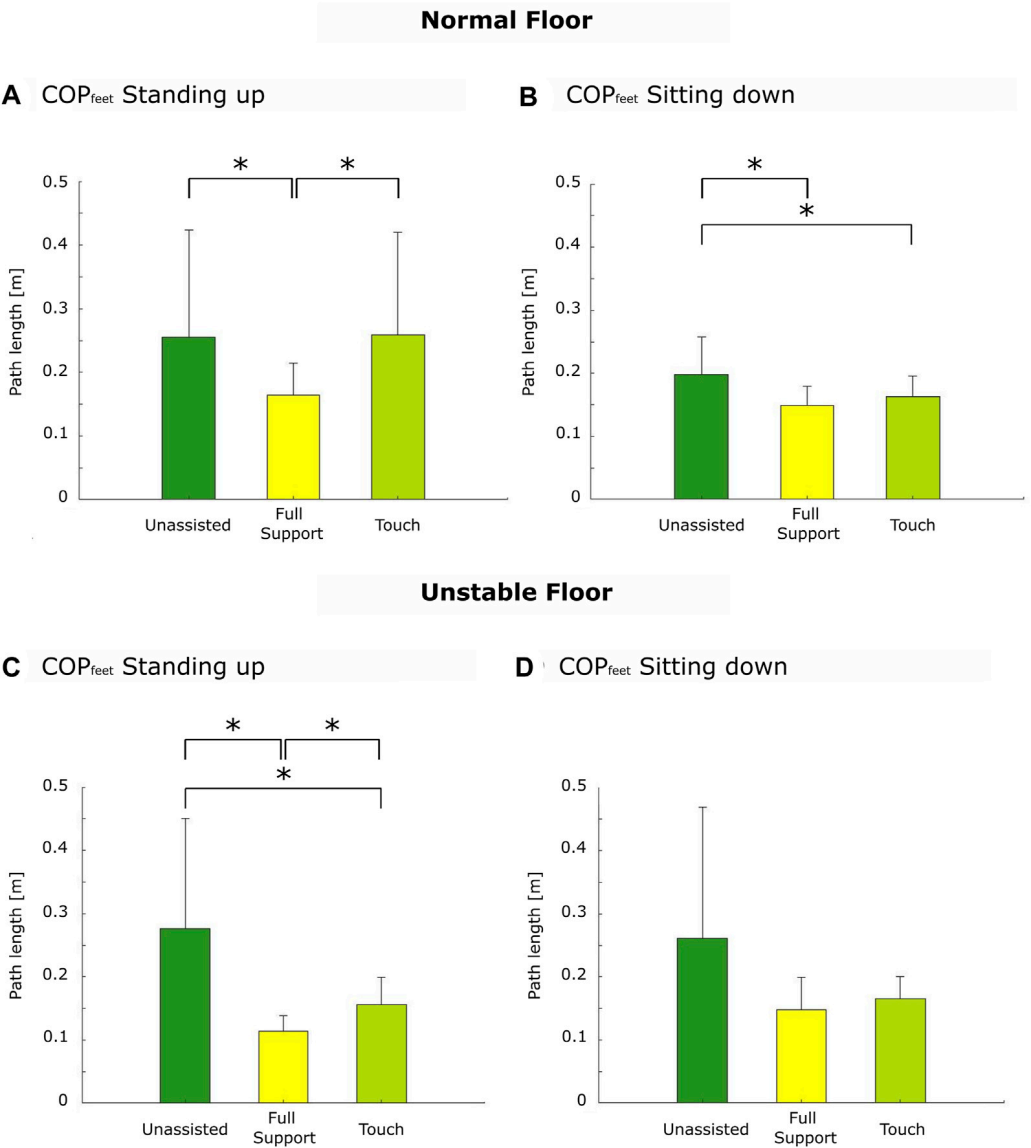
Center of pressure trajectory length. Standing up under normal floor **(A)** and unstable floor conditions **(C)** and sitting down under normal **(B)** and unstable floor conditions **(D)**. The asterisk depicts post hoc significant differences with *p* ≤ 0.05 (Holm–Bonferroni corrected).

During standing up, support conditions affected both measures of stability, namely, COP_feet_ trajectory length and sway area, similarly during both floor conditions, with full support showing increased stability. For COP_feet_ length, there were significant differences in the influence of the support conditions on the COP_feet_ length under normal (*p* < 0.01) and unstable floor conditions (*p* < 0.01). COP_feet_ length was reduced under full support compared to touch by 39.5% (*p* = 0.01; *d* = −0.75) and compared to unassisted conditions by 39.5% (*p* = 0.02; *d* = −0.69) during both normal (Figure 3A) and unstable floor conditions. Here, COP_feet_ length was reduced under full support compared to touch by 31.3% (*p* < 0.01; *d* = −1.15) and compared to unassisted by 70.7% (*p* < 0.01; *d* = −0.62; Figure 3C). The average COP_feet_ trajectory over all participants during standing up on the normal and unstable floors is provided in Supplementary Figure S1.

**FIGURE 4:**
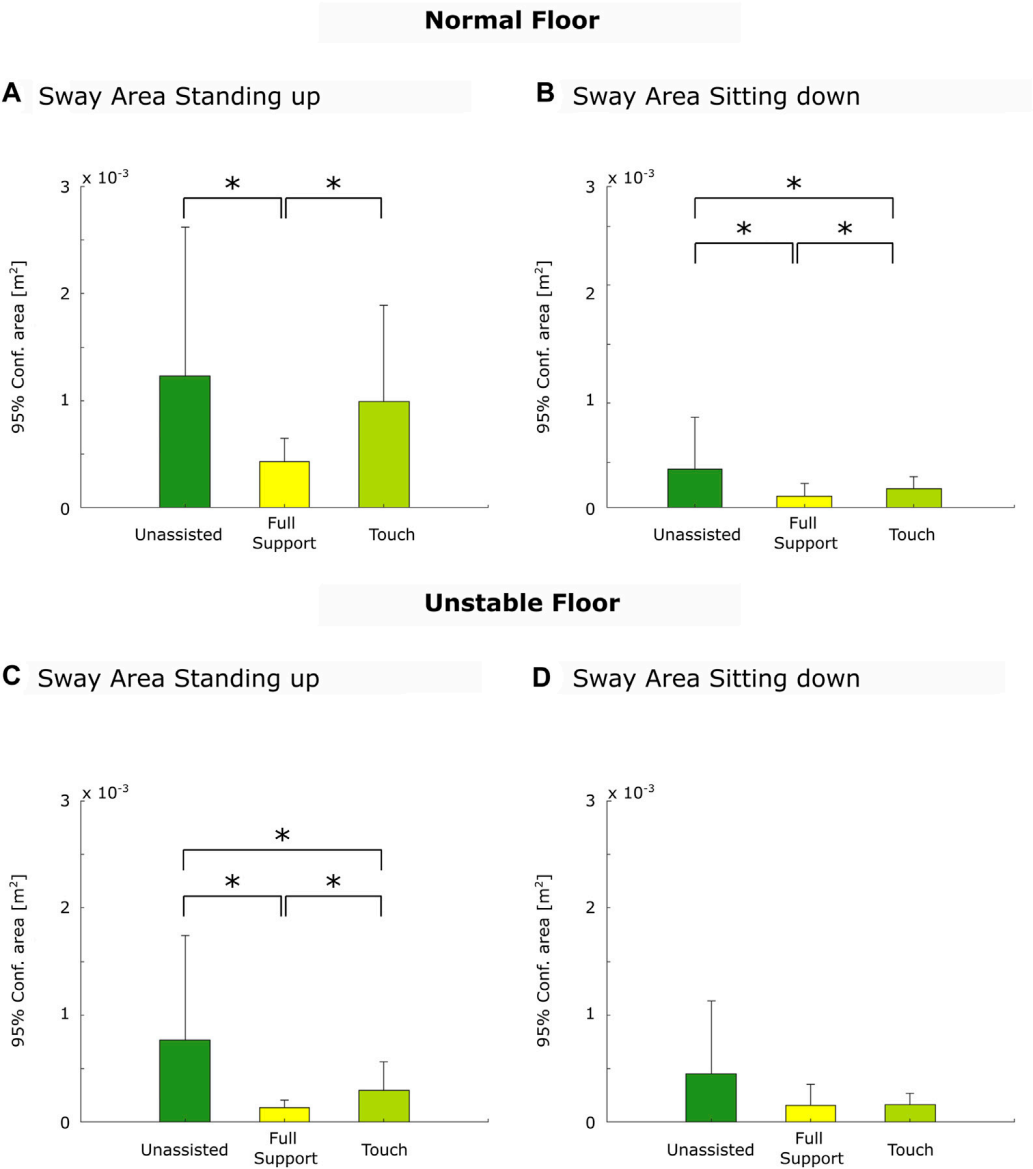
95% confidence ellipse (sway) area. Standing up under normal floor **(A)** and unstable floor conditions **(C)** and sitting down under normal **(B)** and unstable floor conditions **(D)**. The asterisk depicts post hoc significant differences with *p* ≤ 0.05 (Holm–Bonferroni corrected).

For the COP_feet_ sway area, there were significant differences in the influence of the support conditions under normal (*p* = 0.02) and unstable floor conditions (*p* = 0.01). Sway area was reduced under full support compared to touch by 60% (*p* = 0.01; *d* = −0.77) and compared to the unassisted condition by 66.7% (*p* = 0.02; *d* = −0.672) during both normal (Figure 4A) and unstable floor conditions. Here, sway area was reduced under full support compared to touch by 66.7% (*p* = 0.02; *d* = −0.76) and compared to unassisted by 87.5% (*p* = 0.01; *d* = −0.82; Figure 4C).

In summary, during standing up, the highest movement stability was found during full support on the normal and unstable floors.

During sitting down, support conditions affected both measures of stability similarly only on the normal floor, with full support showing increased stability. For COP_feet_ length, there were significant differences in the influence of support on COP_feet_ length under normal (*p* < 0.01) and unstable floor conditions (*p* = 0.03). COP_feet_ length was reduced under full support compared to unassisted by 25% (*p* < 0.01; *d* = −1.06) and reduced under touch compared to unassisted by 20% (*p* = 0.02; *d* = −0.74) on the normal floor (Figure 3B). COP_feet_ length did not significantly differ under unstable floor conditions because of the Holm–Bonferroni-corrected *p*-values for pairwise t-tests. However, tendencies appeared toward reduced COP_feet_ length under full support compared to touch by 11.8% (*p* = 0.06; *d* = −0.37) and compared to unassisted by 42.3% (*p* = 0.02; *d* = −0.72; Figure 3D).

For the COP_feet_ sway area, there were significant differences in the influence of support under normal (*p* < 0.01; Figure 4B) but not under unstable floor conditions (*p* = 0.08; Figure 4D). Sway area was reduced under full support compared to touch by 33.3% (*p* = 0.02; *d* = −0.61) and compared to unassisted by 50% (*p* < 0.01; *d* = −0.94) and under touch compared to unassisted by 25% (*p* = 0.02; *d* = −0.63) on the normal floor.

In summary, during sitting down, the highest stability was also found during full support on the normal floor but not on the unstable floor.

### Influence of Support on Posture After Standing up

**FIGURE 5:**
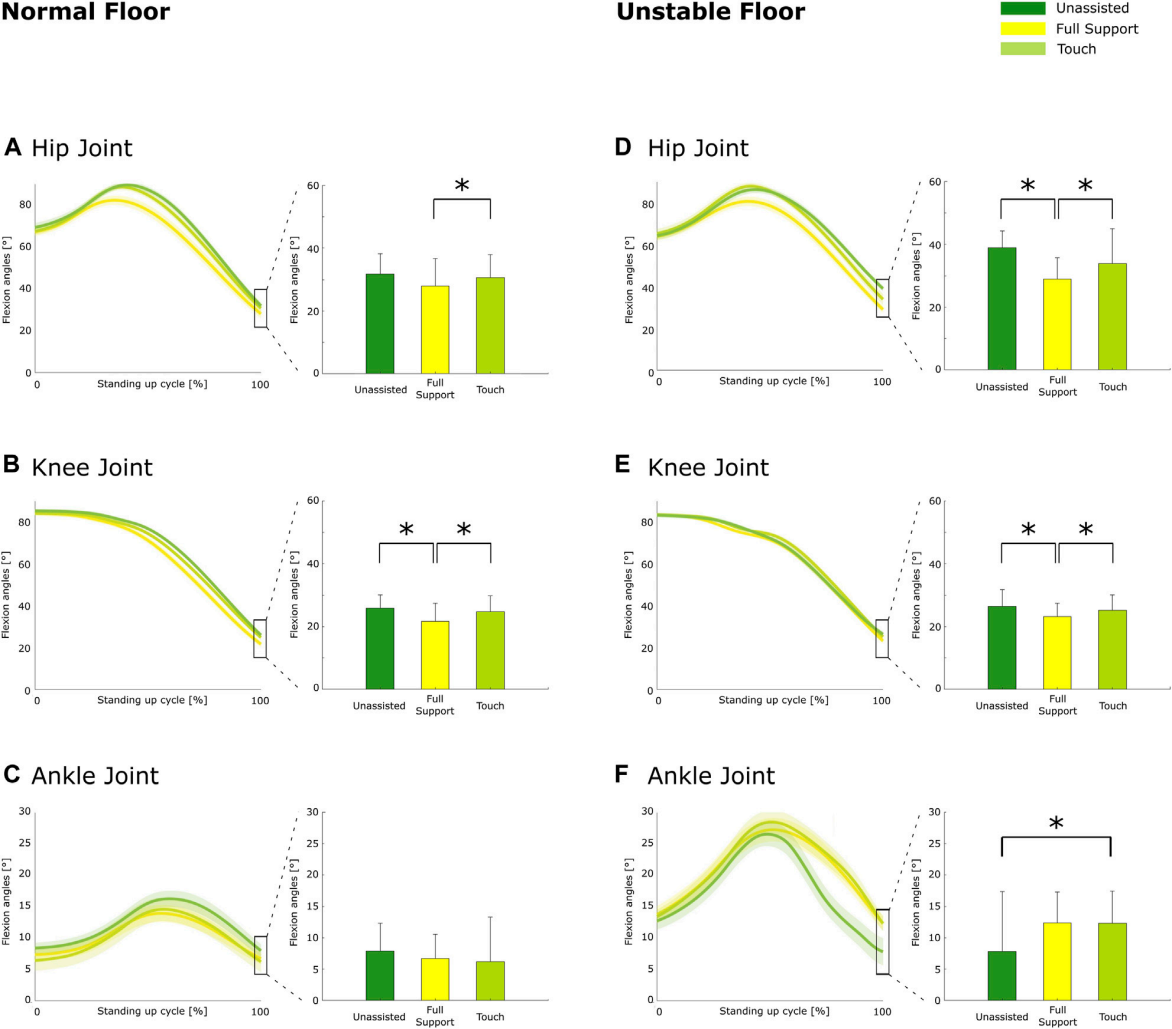
Sagittal plane kinematics at a stable stance on the normal floor for the hip **(A)**, knee **(B)**, and ankle **(C)** and on the unstable floor for the hip **(D)**, knee **(E)**, and ankle **(F)**. On the left side, motion-normalized trajectories are shown with values at a stable stance on the right. Group averages are shown with the bold line, and shaded areas indicate the standard deviations. The bar represents the mean value, the error bar represents the standard deviations of sagittal joint angles at a stable stance, and the asterisk depicts significant differences with *p* ≤ 0.05 (Holm–Bonferroni corrected).

On the normal floor, the support conditions affected the posture at a stable stance at the hip (*p* < 0.01) and knee (*p* < 0.01) but not at the ankle joint (*p* = 0.38; Figure 5C). Under full support, the participants stood with reduced hip flexion by 11.7% (*p* < 0.01; *d* = −0.47) and knee flexion by 16.2% (*p* < 0.01; *d* = −0.82) compared to the unassisted condition (Figure 5A). In addition, knee flexion was reduced under full support compared to touch by 12.5% (*p* = 0.01; *d* = −0.58; Figure 5B).

On the unstable floor, the support conditions affected the posture at a stable stance at the hip (*p* < 0.01), knee (*p* < 0.01), and ankle joint (*p* = 0.02). Under full support, the participants stood with reduced hip flexion by 25.6% (*p* < 0.01; *d* = −0.52; Figure 5D) and reduced knee flexion by 12.4% (*p* < 0.01; d = −0.67; Figure 5E) compared to the unassisted condition. Owing to Holm–Bonferroni-corrected *p*-values, it appeared that ankle dorsiflexion tends to be increased under full support by 57.7% (*p* = 0.02; *d* = 0.55) compared to the unassisted condition (Figure 5F).

## Discussion

In the field of fall prevention, it is still unsolved how the support offered by an assistive device enhances individual movement stability and reduces the risk of falling (Komisar et al., 2019). This is even more important as recent studies have indicated the usage of assistive devices as a fall risk for older individuals (Hefflin et al., 2004; Deandrea et al., 2010). Previous studies have described various movement alterations during assisted movement, such as increased forward-leaning and leg unloading, that could contribute to unstable movement situations (Carey and Crompton 2005; Drzał-Grabiec et al., 2013; Boyer et al., 2017). This is the first study to evaluate the role of rollator support during both standing up and sitting down, with different levels of support and an unstable floor condition. Our results indicated that young, able-bodied individuals are more stable using the rollator for full support: they need less time to prepare for the shift of their COM before seat-off, and because of that, they can realize earlier seat-off, can stabilize their COP_feet_ better during standing up as well as sitting down, and can stand in a more stable—upright—position after standing up.

Considering the influence of support on the spatiotemporal parameters, it was found that full support and touch led to an earlier seat-off compared to unassisted on the normal floor and full support resulted in an earlier seat-off during standing up compared to the touch and unassisted conditions on the unstable floor. Hence, individuals can realize the forward shift of the COM and the lift of the buttocks from the chair earlier when standing up with full support, especially on an unstable floor. On the normal floor, both support conditions enabled an earlier seat-off compared to the unassisted condition. However, the shift in seat-off timing indicates that during full support, the participants could better organize the combined trunk motion and leg joint coordination. As the seat-off phase is considered highly relevant for the stable performance of chair rise (Schenkman et al., 1990; Roebroeck et al., 1994), it is assumed that earlier seat-off enables a more stable performance during standing up because of the better-coordinated interplay of the upper and lower body (Geravand et al., 2017). However, the earlier seat-off did not affect the total standing up duration in favor of full support over the touch and unassisted conditions, which is interesting as enhanced performance of chair rise abilities is often assessed by a reduced overall movement duration (Guralnik et al., 1994; Sütcü et al., 2019).

When considering movement stability, in standing up, it was found that movement stability seemed to be enhanced during full support compared to the other conditions, as represented by reduced COP_feet_ trajectory lengths and a reduced sway area. During sitting down, movement stability was also found to be increased during full support compared to unassisted and touch and during touch compared to unassisted. Because people with lower postural stability often have more problems regulating their COM above their feet, they have larger COP variability during standing and moving (Kędziorek and Błażkiewicz, 2020). Indeed, increased COP trajectory length and sway area are associated with fall risk (Takagi et al., 1985; Norris et al., 2005; Merlo et al., 2012). Our findings indicate that full rollator support shows the potential to improve movement stability during standing up over the other conditions and that both support conditions provide higher movement stability during sitting down compared to the unassisted performance when the handle height is individually well adjusted and positioned in young individuals. During sitting down, it seems that the touch condition provides a sufficient sensorimotor input to perform the sitting down movement with movement stability that is enhanced in the unassisted condition but reduced in the full support condition in terms of sway area. This is in accordance with other studies investigating light haptic cues to improve balance and movement stability in assisted walking (Dickstein et al., 2003; Martinelli et al., 2015; Costamagna et al., 2019; Oates et al., 2020) and shows that not only full rollator support leads to increased movement stability.

The analysis of the sagittal joint kinematics at a stable stance after standing up showed that individuals stand most upright when applying full support to the rollator during standing up. This was concluded as the individuals stood with more extended knee and hip joints in the full support condition compared to the touch and unassisted conditions on the normal and unstable floors. This indicates that the stance was more stable when performing under full support as they needed less co-contracted and co-activated muscles to maintain this stable position, which is needed to support the increased joint flexion of the legs (Bouchouras et al., 2015). Although the absolute hip and knee flexion angles at a stable stance did not range widely between the support conditions (range 3°–10°), medium or large effect sizes (≥0.5) were found for all pairwise comparisons. This implies that the differences are practically meaningful toward full support, leading to the least flexed hip and knee joints at a stable stance (Cohen 1992). These findings are in contrast to the results of recent studies, describing greater forward trunk-leaning along with increased hip and knee flexion during assisted versus unassisted rollator walking in older persons (Carey and Crompton 2005; Drzał-Grabiec et al., 2013). However, the forward-leaning phenomenon could also be induced by variations of handle height (Choi et al., 2015). Choi and others (2015) showed that forward trunk-leaning is increased when setting the handle height at 48% body height compared to setting the handles at 55% body height. Although we defined the handle height at the height of the distal radius head, it resulted in an average handle height of 49.3% body height. The reason that this rather low handle height did not lead to increased forward trunk-leaning could be caused by the fact that rollator handles were held at the side directly against the body during a stance and not in front of it as it is common during assisted walking. Due to this influence of handle positioning, it cannot generally be stated that assistance to a rollator leads to forward trunk-leaning when receiving full support and that generally young participants benefit from rollator assistance in terms of movement stability. These findings let us assume that some individuals with stability issues could benefit from just a touch of the handles of that rollator as this could stimulate their own stability mechanisms better than when they were fully supported. This knowledge could result in instructions for older persons to hold the handles differently and for the design of rollators to have more light-weight friendlier-to-use rollators compared to rollators for users that need higher body weight support.

There are some limiting aspects to our study. The rollator simulator differs from a rollator used in real life. The device is heavier and larger than a sturdier rollator, which has the ethical advantage of preventing the rollator from tipping over. However, it prevents us from studying real-world falls as a result of tipping, and the psychological effect on movement because of fear of tipping and falling is unknown. However, owing to its degrees of freedom, it allows a wide range of individual adjustments (e.g., handle height and handle positioning) and has the potential to strongly control any provided assistance during different movements and its implementation in a human movement laboratory.

Next, the analysis of the COP_feet_ alone does not represent the COP of the combined system of the rollator and the user. However, as during the unassisted condition the rollator simulator was not used, the analysis of the COP_feet_ provides a consistent insight of the effort of the human body to regulate the COM within the base of support during the transition phase from seat-off to a stable stance for the comparison of the two support conditions and the unassisted condition. As the COP reflects the neuromuscular response to control the COM within the base of support (Sloot et al., 2020; Richmond et al., 2021), the findings of the study help in understanding how the body constrains the COM within the base of support during assisted and unassisted standing up and sitting down. This was shown in this study by the reduced COP_feet_ length and sway area with increasing support, implying that less effort of an individual was needed to control the COM within the base of support.

To improve the analysis of the combined system of user and rollator simulator, upcoming work targets to develop a better model for both COP and BOS calculation for the combined system of the user and the rollator simulator. Although humans are not able to move the COP to or beyond the edges of the base of support formed by our feet (Sloot et al., 2020) and it is assumed that the COP cannot be shifted to the outer edges of the rollator, such a model will help in providing more sophisticated analyses of movement stability during assisted movements and the inclusion of various other accepted balance metrics. In this study, the combined 2D horizontal COP_feet_ and sway area were analyzed to give a baseline result of the influence of support on the standing up and sitting down movement. Beforehand isolated analysis of the anterior–posterior and medio-lateral COP_feet_ components revealed the same finding as the combined COP_feet_.

Following studies will focus on a more heterogeneous population. For better generalizability of the data, a larger sample size with a larger variation of the physical state of the participants would have helped in obtaining results that reflect the general population. With the recruitment of the participants affiliated to both involved institutions, we rather ended up with a sample that had an active lifestyle. In addition, this population did not need nor was used to rollator assistance. However, examining younger individuals enables a relevant baseline assessment for the role of support as those participants are not influenced by the age-related decline of physical activities nor have any experience with rollator interaction in daily life. Hence, the next step is to perform the same measurements in older individuals who already use rollators throughout their daily life because of age-related muscular weakness and stability deficiencies. Therein, it would be also of important relevance to analyze left–right differences in the legs to better operationalize the individual deficiencies that could lead to falls. Moreover, the operationalization of events that lead to falls is very difficult. In this study, an unstable situation was induced by balance pads with a compliant surface under each foot. As the participants could see those pads and could perform two familiarization trials before performing the task, these could have withdrawn the intrinsic or extrinsic non-anticipatory nature in which falls occur in real-life scenarios (Masud and Morris 2001).

## Conclusion

This study has shown that full rollator support seems to improve movement stability in young, able-bodied individuals most in terms of less preparation time needed for realizing seat-off, improved COP_feet_ control and sway area during standing up and sitting down, and a more upright posture when reaching a stable stance. These improvements were similar when the balance was perturbed in the unstable floor condition. The differences between full support and touch show that the improvement in balance was not just caused by the extra proprioceptive information provided by solely touching the handles with a haptic cue. In addition, the results indicate that full leaning on an assistive device in itself does not lead to a hunched body posture during sit-to-stand transfers as sometimes suggested for walking, which could be related to handle height and positioning. In contrast, the reasons for this phenomenon could be caused by improper handle adjustment and positioning. To get a clearer picture of the effects of assistive support and fall prevention, comprehensive biomechanical analyses should be conducted in older individuals that already use an assistive device during their daily life.

## Data Availability

The raw data supporting the conclusion of this article will be made available by the authors, without undue reservation.

## Supplementary Material

Supplementary Figure S1 Mean combined anterior–posterior and medio-lateral center of pressure trajectories during standing up on normal **(A)** and unstable **(B)** floors. Zero-point (starting point) was normalized to 0/0 in each trial for every individual.
