# Supplementary figures and images for: Young Individuals Are More Stable and Stand More Upright When Using Rollator Assistance During Standing up and Sitting Down
Source: Front Bioeng Biotechnol. 2022 Jul 11;10:876349. doi: 10.3389/fbioe.2022.876349 (PMC9309795; doi:10.3389/fbioe.2022.876349)

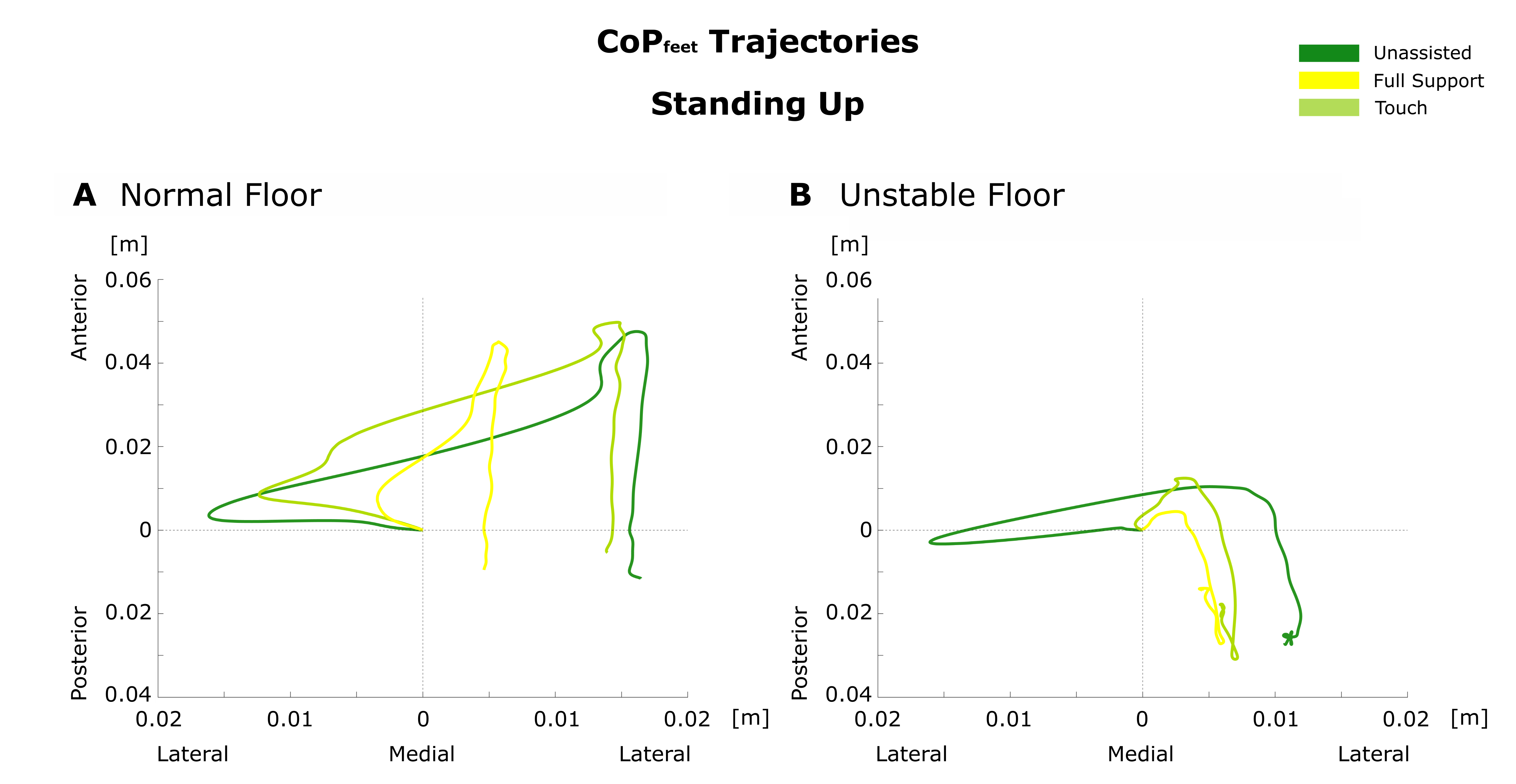

Supplement: Supplementary file 1 [file Image1.TIFF]
